# Germination of bean seeds (*Vigna unguiculata* L. Walp.) in strong electric fields

**DOI:** 10.1016/j.mex.2023.102490

**Published:** 2023-11-17

**Authors:** Andreas Ries, Juan V. Benítez, Antonio Samudio, Raquel Armoa, Héctor D. Nakayama

**Affiliations:** Centro Multidisciplinario de Investigaciones Tecnológicas (CEMIT), Campus Universitario de la Universidad Nacional de Asunción, Dr. Gaspar Villamayor c/ Dr. Cecilio Baéz, San Lorenzo 111421, Paraguay

**Keywords:** Bean, Germination, Electrofreezing, Seeds, Seed germination in electric fields

## Abstract

This paper presents a tutorial for the germination of bean seeds (*Vigna unguiculata* L. Walp.) in strong electrostatic fields up to 1240 V/cm. The seeds were allowed to germinate under different electric field strengths for 48 h. Although most of such germination experiments did not show any visible effect, the field strength of 945 V/cm strongly increased the seedling's vigor during the early growth stage. In the end, 30 % more yield was obtained from stimulated seeds when compared to the control group. This article postulates for the first time a hypothesis of the mechanism of action of the electric field during germination. In biological cells of any species, water confined between narrow surfaces can undergo a phase transition that shifts its melting point to higher temperatures when an external electric field is applied. This effect has already been known as electrofreezing, and has been confirmed by several experimental and molecular modeling studies. As a consequence, the transport kinetics of molecules across cell organelle membranes might be altered, which in turn leads to different plant properties.

With emphasis on the presented method, this work reports:

• An inexpensive electric circuit for the generation of strong electric fields
• Instructions regarding the setup and operation of an adequate germination chamber

## Related research

G. Ebner, H. Schürch. Method of Breeding Fish. United States Patent No.: 5048458, filed on 19/06/1989, and: G. Ebner, H. Schürch. Methode zur Behandlung von biologischem Material, European Patent No.: EP0791651, filed on 22/01/1997, (In German language).

## Graphical abstract

**Figure:**
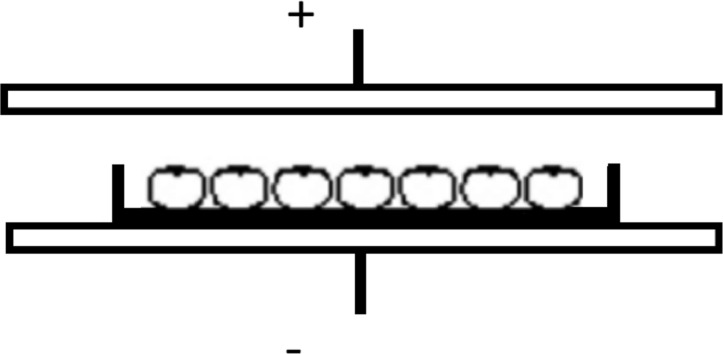

Specifications table

**Table:** 

Subject area:	Agricultural and Biological Sciences
More specific subject area:	Plant Science
Name of your method:	Seed germination in electric fields
Name and reference of original method:	Method of Breeding Fish. United States Patent No.: 5048,458
Resource availability:	not applicable

## Introduction

Electric field effects on plants have been investigated since the early 1900s by numerous authors. Early publications from 1898 to 1927 [1,2] claim an enhanced plant growth caused by a weak electric field.

Later in 1989, the patent literature mentioned the effects of electric fields on biological material. For instance, the patent entitled “Improved method for breeding fish” [3,4] describes how fish (trout, Latin name: Salmo trutta) can develop particularly well if their inseminated eggs are previously exposed to an electrostatic field.

Compared to untreated fish eggs, the hatching rate increased from 100 % to 300 %. The fish were more agile and vital, highly resistant to disease, and grew in size and weight much faster and reached adulthood earlier.

Another patent [5] entitled “Method for the treatment of biological material” describes the effect of electrostatic fields on various life forms such as bacteria and seeds (wheat, corn, etc.). Here, plant seeds were exposed to an electric field only during the germination phase, which means for a few days; then the plants grew without artificial influences.

Such literature references raise a general question of whether cells can somehow feel electric fields. Nowadays it can be indeed considered proven that cells can detect static electric fields. Zhao et al. [6] reported that the orientation of cell division is driven by applied electric fields of 150 V/m. Cultured human corneal epithelial cells divide preferentially with a cleavage plane perpendicular to the electric field vector.

Also very interesting is the fact that tomatoes remain fresh longer when stored in an electric field of 2 kV/cm [7]. Algae of the species *Chlorella vulgaris,* treated in an electrostatic field of 2.7 kV/cm can accelerate or retard growth depending on the treatment time [8].

Investigation into electric field effects is still a minor research field in plant science and most studies deal with seed germination (e.g. review by Schmiedchen [9]). For instance, Moon and Chung reported accelerated germination of tomato seeds in alternating electric and magnetic fields [10].

Mahmood et al. [11] exposed wet and dry seeds of *Pisum sativum* (pea) to an electric field of 0.25–1.5 kV/m and 25–125 kV/m for 8 min. This is a quite short period, insufficient to reach a relevant degree of germination. The seeds were then allowed to germinate without any applied electric field. On the sixth day, the length of stems and roots was measured. In most cases, the electric field caused an increased stem and root length.

An improved germination rate and accelerated plant growth were found for Radish when its seeds were subjected to an electric field of 2.5 kV/m for only 3 h per day. The total stimulation time in this experiment was 7 days [12].

Field effects on germination are not always reported as positive. According to Isobe [13], dried seeds of “Japanese morning glory” (*Pharbitis nil*), an ornamental plant, were treated in a static electric field of 500 kV/m for 60 min. Then, the seeds were allowed to germinate on moist filter paper in a temperature-controlled chamber at 25 °C for 48 h under fluorescent lamps. The germination rate of treated seeds was 44 %, while that of untreated seeds was 84 %.

Shen et al. [14] exposed *Sorbus pohuashanesis* seeds to electrostatic fields of 100 to 250 kV/m for periods between 5 min and 40 min. After treatment, the time to onset of germination was shortened. The authors concluded that electrostatic field treatment can increase seedling height, significantly affect superoxide dismutase activity, and significantly improve leaf soluble protein content, total soluble sugar content, and total leaf chlorophyll. That is, such treatment is an effective way to improve seed viability and germination, seedling resistance, photosynthetic capacity, and subsequent seedling growth.

Arruda-Neto et al. [15] reported the combined effects of an electrostatic field and ionizing radiation on cyanobacteria (*Microcystis panniformis*), *Candida albicans,* and human lung cells. These biological materials were exposed to gamma radiation at a dose of 5 kGy, and then stored in an electrostatic field (20 V/cm for cyanobacteria, 180 V/cm for *Candida albicans,* and 1250 V/cm for human cells). The authors found that the application of electrostatic fields greatly increases cell death. Even weak fields interfere with DNA repair mechanisms.

In this article, we focus on the methodology of seed treatments as described in the early patent literature [3]. Bean seeds were allowed to germinate inside a strong electric field. We confirm the existence of accelerated initial growth and propose a new mechanism of field action based on molecular mechanics calculations already published in the literature. This method differs from traditional priming methods, in that the seeds are not just treated for a short time period by a field (electric or magnetic or both). To the best of our knowledge, this is the first study reporting the action of an electrostatic field during the whole germination process of seeds. So this process is certainly more time-consuming. It has the advantage that no chemical products are required, consequently, no residues could remain in the seeds or harm the environment.

## Method details

### Seeds

Cowpea (*Vigna unguiculata* L. Walp.) is an annual bean plant that completes its life cycle within one growing season and then dies. Pods mature in about 90–125 days after sowing. Specifically in Paraguay, the plant is also known as "kumanda", variety “Pytã'i” in the local Guarani language. It is an edible legume cultivated in all tropical and subtropical regions of the world. It has medium-sized, dark red grains. The crop is typically harvested in the first half of November.

These seeds were chosen because it is a species of easy propagation, presenting uniform characteristics in terms of seed germination. In addition, its culinary quality is very good. For that reason, this crop is widely produced by small farmers in the region for the local market.

**Fig. 1:**
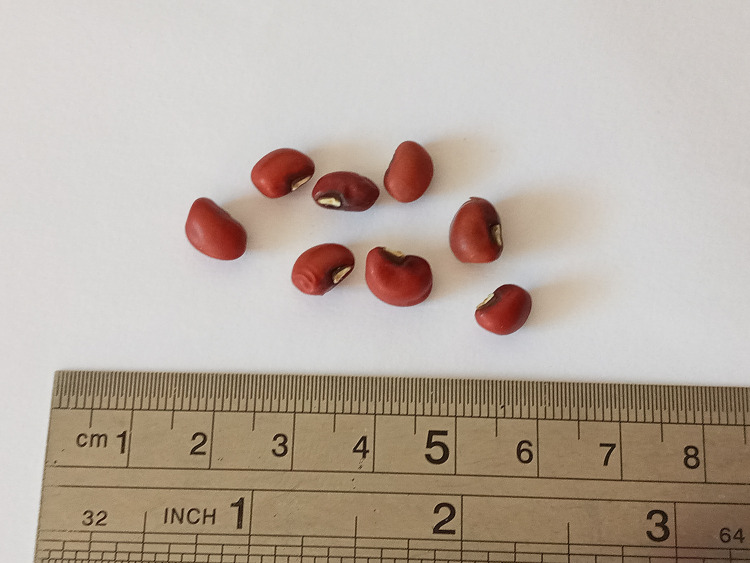
Dark red bean seeds.

A local farmer supplied bean seeds. These were harvested in 2021, then dried to 13 % humidity, and stored in glass jars with screw caps in a warehouse with constant humidity and temperature. Seeds were washed 4 times with sterile water for 4 min prior to germination to remove possible bacterial contaminations. During this washing time, seeds already absorb a significant quantity of water (reaching a moisture content of 90 %). Fig. 1 shows a photograph of dry bean seeds used in this work.

### Germination and growth

For germination, 20 washed seeds were placed on wet filter paper in a Petri dish and kept for 48 h inside the electric field. Seeds that did not germinate within this period were discharged. Next, the vigor of germinated seeds was observed for 1 week in a plastic dish.

In the case of successful stimulation (shown by a change in vigor), 14 plantlets were grown outdoors, together with an equal number of untreated plantlets that served as a control group. These plants were considered for the analysis of yield. No fertilizer was used, but the plants were treated once a week with cypermethrin against aphids. After 12 weeks of outdoor growth, the complete harvest occurred within 3 weeks.

### Experimental apparatus for generating the electric field

For generating strong DC electric fields, a five-stage Cockcroft-Walton [16], [17], [18] voltage multiplier circuit was chosen as a generator; it is built from a cascade of capacitors and diodes. Each stage consists of two capacitors and two diodes. The circuit is connected directly to 220 V AC (alternating current), without the use of a transformer.

**Fig. 2:**
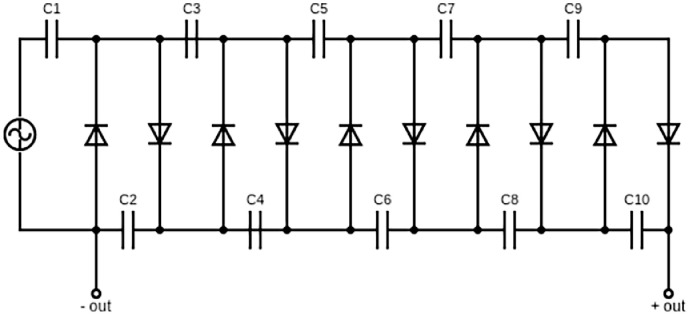
Circuit diagram of the five-staged Cockcroft-Walton voltage multiplier.

The circuit schematic is shown in Fig. 2. A detailed explanation of its operation principle from the viewpoint of electrical engineering is given elsewhere [19], [20], [21], [22], [23], [24].

The output voltage is the sum of the voltages across C2, C4, C6, C8, and C10. Depending on the number of diodes and capacitors, the cascade provides a theoretically arbitrary output voltage. Under the no-load condition, the output voltage is equal to the peak voltage at the AC input, multiplied by the number of diodes. However, in practice, a limit is set by two facts: there are switching losses in the diodes and the capacitors are connected in series. As the number of capacitors increases, the total capacitance of the cascade becomes smaller and smaller. As a result, the output voltage eventually collapses as soon as a minimal current is drawn.

Fortunately, for germination in an electric field, no electric current is actively drawn; it is only necessary to compensate for diode and capacitance losses as well as the tiny leakage current between the metal plates due to ionization of the air.

An advantage of the series connection of the capacitors is that, despite the high output voltage, each capacitor only needs to withstand twice the peak voltage; this means that, with an effective input voltage of 220 V, the peak voltage is the square root of 2 multiplied by the effective value, i.e. 325 V (twice the peak voltage is 650 V). For this to occur the diodes must withstand the same voltage. A good rule of thumb is to select a capacitor whose allowable operating voltage is approximately twice the actual applied peak voltage (1.3 kV).

In this work, all capacitors were 400 nF ceramic with a nominal voltage of 2 kV. All diodes were model UF4007, with a maximum peak repetitive reverse voltage of 1000 V, a maximum average rectified output current of 1 A, and a reverse recovery time of 75 ns.

Experimentally, we measured the following voltages across the capacitors C2, C4, C6, C8, and C10: 625 V, 572 V, 524 V, 488 V, and 456 V. This leads to a total output voltage of around 2.5 kV; it can be seen that with every additional stage, the increase in total output voltage becomes lower.

Finally, the electric field strength (total voltage/plate distance) is adjusted by varying the distance between the stainless-steel plates. In this study, different plastic parts from water pipes were used as distance holders. The following four obtained field strengths were employed: 470 V/cm, 763 V/cm, 945 V/cm, and 1238 V/cm.

**Fig. 3:**
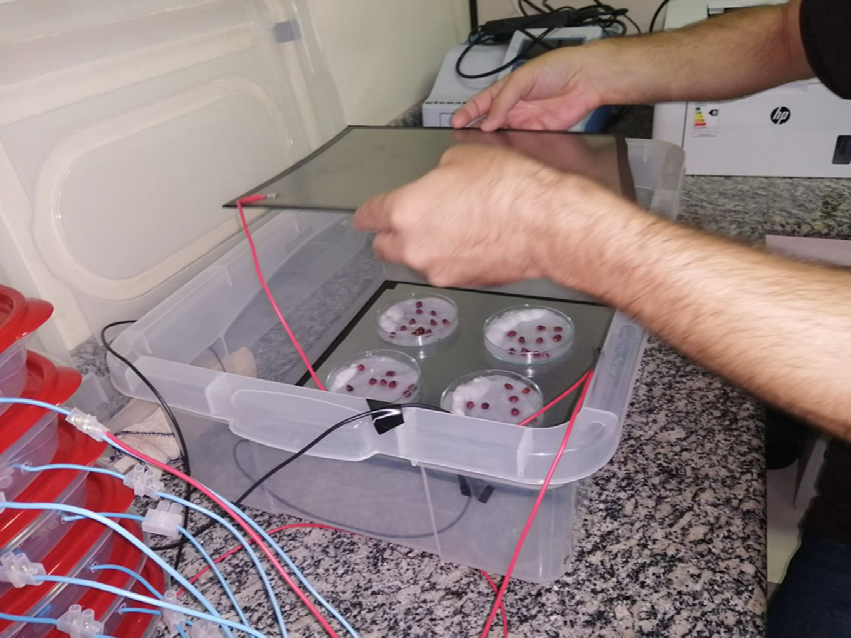
Seed samples in Petri dishes on wet filter paper between stainless steel plates.

Seed samples are then positioned between two steel plates, according to Fig. 3. The negative pole of the output voltage was connected to the bottom plate, while the top plate was positively charged.

Under these field conditions, no ozone formation or silent discharges inside the device were detected.

Finally, we would like to advise the reader to avoid any black plastic parts as distance holders. Very frequently, the black color is caused by carbon black, which is a filler added to the polymer. Besides being a pigment, it introduces conductivity to the plastic, thus, serving as an antistatic agent. Although the conductivity of such plastics is still low, under those high voltages, the capacitor bank is already discharged significantly. We observed experimentally a drop in the output voltage of around 1 kV when black plastic parts served as a distance holder.

### Method validation

After 48 h on wet filter paper, independent of the applied field strength, the observed germination rate was always higher than 90 %. This was expected since beans do not possess a tough seed coat, nor do they have any dormancy problems.

**Fig. 4:**
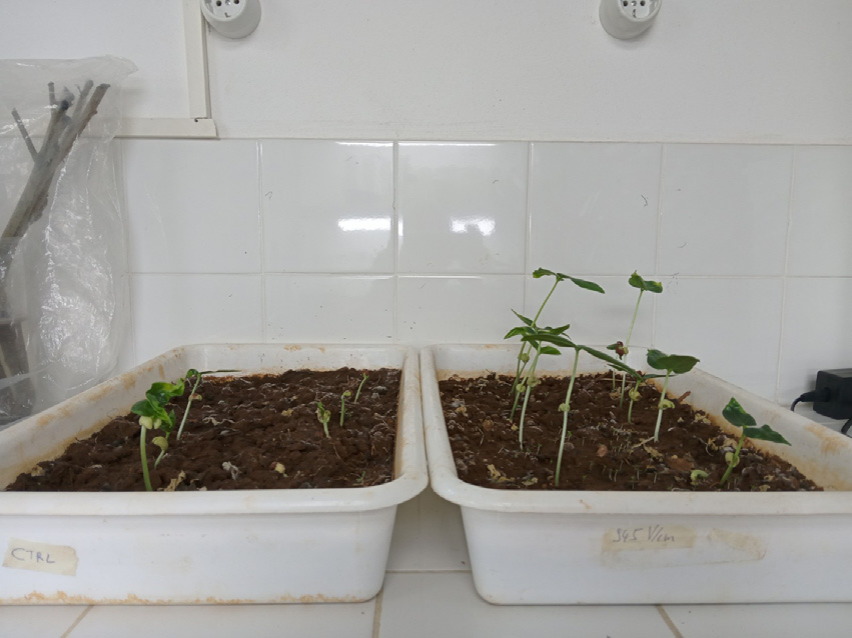
Bean seedlings after 3 days in a plastic dish. Control (left) and germinated in an electric field of 945 V/cm (right).

**Fig. 5:**
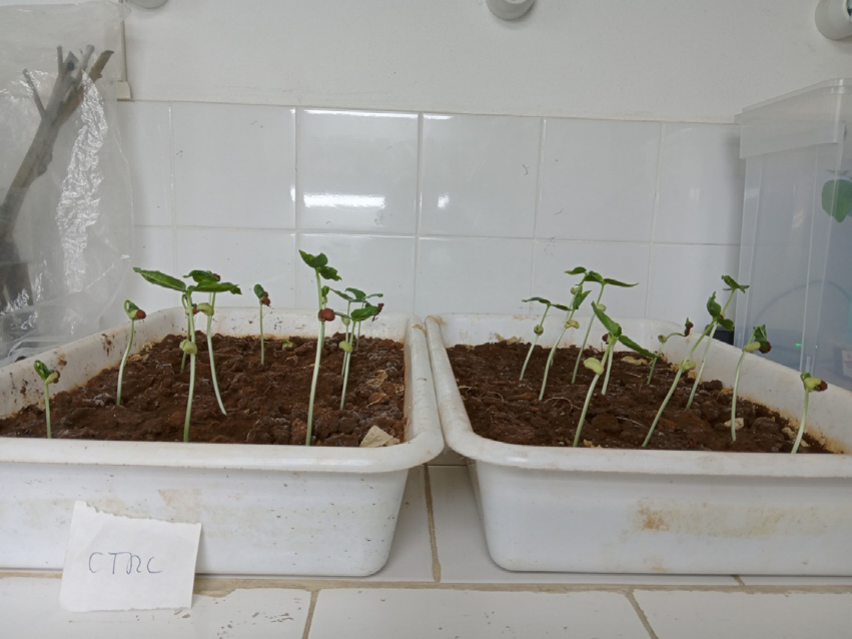
Bean seedlings after 5 days in a plastic dish. Control (left) and germinated in an electric field of 763 V/cm (right).

Early growth monitoring in plastic dishes revealed that only the field strength of 945 V/cm affected the seedling vigor. Fig. 4 shows the difference in vigor for bean seedlings on the third day. For comparison, the field condition of 763 V/cm is displayed in Fig. 5; no significant difference in plant size is visible. Equally, no change in vigor was noted for the remaining field conditions (results not shown). This change in vigor is interpreted as a successful stimulation, as observed in stimulation experiments by others [11].

Next, fourteen plants (14 stimulated and 14 control) were allowed to grow on farmland; with increasing lifetime, the sizes of the plants became more and more equal.

**Fig. 6:**
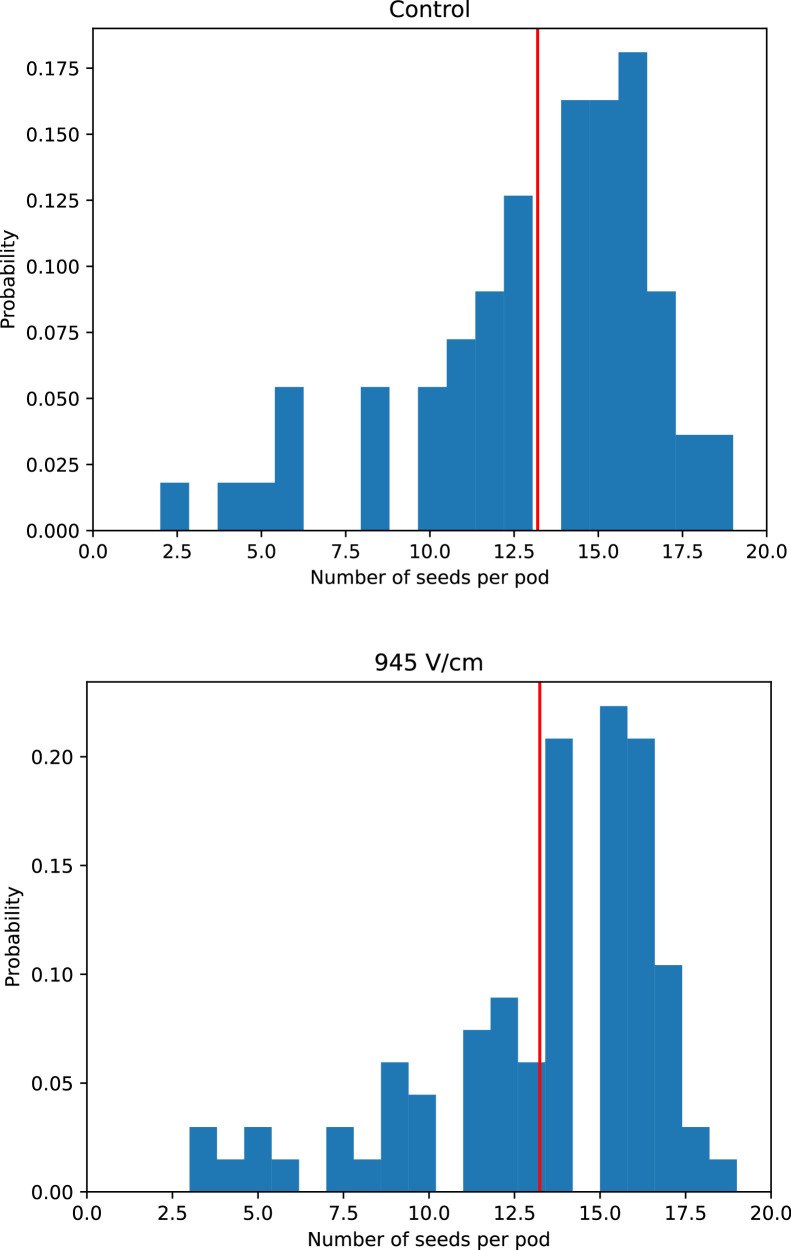
Histogram plot: Number of bean seeds per pod for control (top), and germinated in an electric field of 945 V/cm (bottom). The red vertical lines indicate the mean values of seeds per pod; the bin size was set to two.

All harvesting took place within the last three weeks of the plants' life cycle. The 14 stimulated bean plants yielded 85 pods, while only 65 pods were harvested from the control group. To accurately compare yields, it is necessary to analyze the number of seeds in the pods. Fig. 6 shows the corresponding histogram plots.

Surprisingly, the electric field does not influence the mean value of seeds inside a pod; for both conditions that value was found exactly as 13.2 seeds per pod when rounding to one figure after decimal point. Histograms also have a quite similar shape. Consequently, any increase in yield is due to a larger number of harvested pods. It is now possible to calculate the yield for both cases by multiplying the number of harvested pods with the mean value of seeds inside. Setting the yield of the control group to 100 %, the stimulated plants provided 30 % more seeds in total.

Another way to determine the yield is just by weighing the harvested bean seeds from both groups (118 g from the control group and 163 g from 954 V/cm). Analogously, the calculation results in 38 % more yield in weight for the stimulated plants. From that, it can be concluded that the stimulated plants certainly do not form smaller seeds than the control group.

**Fig. 7:**
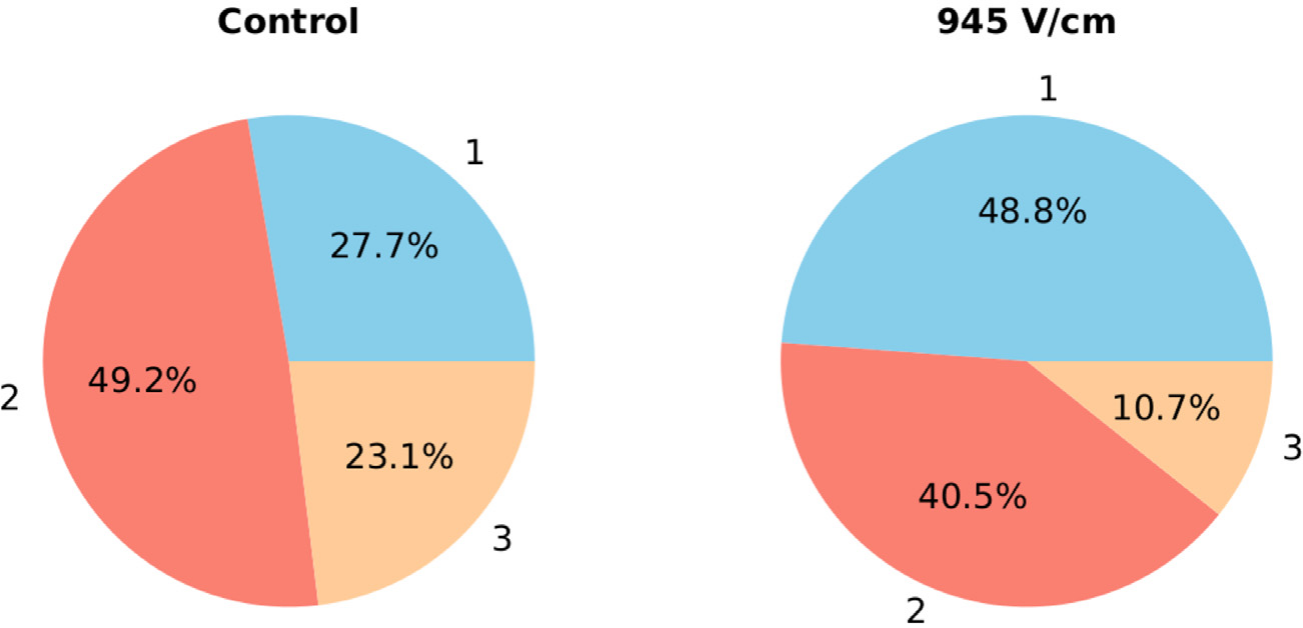
Percentages of pods harvested over the weeks 1, 2, and 3 for control (left), and germinated in an electric field of 945 V/cm (right).

The harvesting dynamic over 3 weeks is significantly different when comparing stimulated and control plants. In the case of stimulation, almost half of the yield had already been obtained during the first week, and after the second one, the harvest was already 90 %. Contrary to this, the control plants produced a more equilibrated harvest, basically one quarter in the first week, half of the yield in the second week, and the remaining quarter in the third week (see Fig. 7).

Despite the abundant evidence presented on the positive effects of pre-treatments of seeds by electric fields, the mechanisms by which these effects are generated have not yet been elucidated.

When carefully reviewing the literature, there are some studies dealing with the modification of the water-to-ice phase transition in dependence on an electric field. These works are not even new, but due to their publication in specialized physics journals, the agricultural science community has not yet noticed that these important findings can be applied to biological systems.

The key idea is the following: When considering an ordinary cup of water inside a strong electric field, the melting temperature of water is not measurably influenced by the field; we always find zero degrees Celsius under standard pressure. However, as soon as the cup size is reduced to a nanometric scale, this picture changes. It has been shown that water confined between two narrow surfaces in the order of nanometers, possesses a melting temperature that is now dependent on an externally applied electric field. Actually, such a field increases the melting temperature of ice, which means liquid water above zero degrees Celsius can be electrofreezed under adequate conditions.

Already in 1998, Stutmann [25] showed that an electric field of approximately 3 V/Angstrom turns water into ice (200 water molecules in a cubic box with sides of 18.16 Angstrom). However, such a high field strength is impossible to achieve in practice.

In 2003, computer simulations by Zangi [26] predicted the possibility that water between two parallel walls with a distance from 1.14 to 1.86 nm would freeze even at 300 Kelvin (approximately 27 °C) and an applied field with a magnitude of 5 V/nm.

In general, the degree of ordering of water molecules decreases as the distance between the two confining walls increases. Freezing to ice is even allowed to occur spontaneously without an electric field provided that water is subjected to an extremely confined state and the water-wall interaction is appropriate (polar walls). Several computational [27], [28], [29], [30] and experimental [31] studies confirm the spontaneous freezing of few-layer ice confined between two parallel plates separated by nanometer gaps.

A very recent study by Khusnutdinoff [32] proves that an electric homogeneous field has a direct impact on water crystallization inside graphene nanotubes. Again, the electric field promotes a structural ordering of water confined between two graphene layers and favors the formation of ice.

In light of the above-quoted literature, the following mechanism of action of the electric field during germination can be postulated: In any cells, we have small, nanometer-sized distances between membranes or cell organelles, which are filled with water. When now due to an external field the water changes its structure from liquid to ice, any transport of molecules through the corresponding membranes can be altered. Thus, the field changes the kinetics of transport processes inside the cell, and this might be the key to understanding accelerated or delayed germination, and enhanced or retarded early growth rate. Also, the fact that in this experimental study, no extremely high fields were applied does not imply that there is no water-to-ice transition in cells. The electric field locally existing between membranes depends much more on the dipole molecules forming these phase boundaries. The role of the externally applied field is much more to polarize, orient and align these dipoles spatially than being the sole cause of the phase transition. In our opinion, this idea should be discussed in the horticulture and biology community.

## Conclusions

The performed experiments demonstrate that germination inside an electrostatic field is a suitable method to influence plants during the germination phase. It was possible to find a condition that had a clear effect on growth in the first weeks. Future research should aim to reproduce the results presented here considering a larger number of plants and real outdoor conditions.

This study reports a single experiment using 20 seeds for stimulation and 20 seeds for control. Only 14, successfully germinated seeds from each group were planted outdoors in order to compare the yield. The objective of this paper is to communicate an adequate condition regarding how an electrostatic field can interfere with the germination process and improve the yield of *Vigna unguiculata* L. Walp, without providing an exhaustive statistical analysis. Having in mind that the yield is influenced by various parameters, such as climate and weather, soil quality, planting density, pest and disease management, weed control, and the age of the employed seeds, any statistical analysis is always biased by external factors the farmer cannot control or parameters that cannot be quantified exactly (such as weed control). Readers should keep in mind that under real farming conditions, the reported improvement in yield may vary.

There remains one fundamental problem to be solved when employing this method for other plants on a large scale: how can we best put germinated seeds into farmland through sawing machines, without damaging the seeds, e.g., without breaking the tiny roots? Once this is solved, there is no obstacle to applying the presented seed treatment commercially.

## Ethics statements

Not applicable.

## CRediT author statement

All authors have contributed equally.

## Declaration of Competing Interest

The authors declare that they have no known competing financial interests or personal relationships that could have appeared to influence the work reported in this paper.

## References

[1] Shibusawa M., Shibata K. (1927). The effect of electric discharges on the rate of growth of plants. J. Inst. Electr. Eng. Jpn..

[2] Hull G.S. (1898). https://archive.org/details/electrohorticult00hullrich/mode/2up.

[3] G. Ebner, H. Schürch, V. Fischzuchtverfahren, European Patent No.: EP0351357, filed on 15/06/1989. (2023) (In German language).

[4] G. Ebner, H. Schürch, Method of breeding fish. United States Patent No.: 5048458, filed on 19/06/1989

[5] G. Ebner, H. Schürch, Methode zur Behandlung von biologischem Material, European Patent No.: EP0791651, filed on 22/01/1997, (In German language)

[6] Zhao M.J., Forrester V., McCaig C.C. (1999). A small, physiological electric field orients cell division. Proc. Natl. Acad. Sci. U. S. A..

[7] Wang Y., Wang B., Li L. (2008). Keeping quality of tomato fruit by high electrostatic field pretreatment during storage. J. Sci. Food Agric..

[8] Nezammahalleh H., Ghanati F., Adams T.A., Nosrati M., Shojaosadati S.A. (2016). Effect of moderate static electric field on the growth and metabolism of Chlorella vulgaris. Bioresour. Technol..

[9] Schmiedchen K., Petri A.K., Driessen S., Bailey W.H. (2018). Systematic review of biological effects of exposure to static electric fields. Part II: invertebrates and plants. Environ. Res..

[10] Moon J.D., Chung H.S. (2000). Acceleration of germination of tomato seed by applying AC electric and magnetic fields. J. Electrost..

[11] Mahmood B., Jaleh S., Yasaman Y. (2014). Study of DC and AC electric field effect on Pisum sativum seeds growth. Eur. Phys. J. Appl. Phys..

[12] Okumura T., Muramoto Y., Shimizu N. (2012). Influence of DC electric field on growth of daikon radish (Raphanus sativus). IEEE Trans. Dielectr. Electr. Insul..

[13] Isobe S., Ishida N., Koizumi M., Kano H., Hazlewood C.F. (1999). Effect of electric field on physical states of cell-associated water in germinating morning glory seeds observed by 1H-NMR. Biochim. Biophys. Acta.

[14] Yang L., Shen H. (2011). Effect of electrostatic field on seed germination and seedling growth of Sorbus pohuashanesis. J. For. Res..

[15] Arruda-Neto J.D., Friedberg E.C., Bittencourt-Oliveira M.C., Cavalcante-Silva E., Schenberg A.C., Rodrigues T.E., Garcia F., Louvison M., Paula C.R., Mesa J., Moron M.M., Maria D.A., Genofre G.C. (2009). Static electric fields interfere in the viability of cells exposed to ionising radiation. Int. J. Radiat. Biol..

[16] Ma Z.W., Su X.D., Lu X.L., Wei Z., Wang J.R., Huang Z.W., Miao T.Y., Su T.L., Yao Z.E. (2016). 250kV 6mA compact Cockcroft-Walton high-voltage power supply. Rev. Sci. Instrum..

[17] He Z.F., Zhang J.L., Liu Y.H., Zhang Y.T., Zhang Y. (2012). Characteristics of a symmetrical Cockcroft-Walton power supply of 50Hz 1.2 MV/50mA. Rev. Sci. Instrum..

[18] Everhart E., Lorrain P. (1953). The Cockcroft-Walton voltage multiplying circuit. Rev. Sci. Instrum..

[19] Fukuyama T., Sugihara K. (2016). Study on operating principle of Cockcroft-Walton circuit to produce plasmas using high-voltage discharge. Plasma Fusion Res..

[20] Quraan M., Zahran A., Herzallah A., Ahmad A. (2020). Design and model of series-connected high-voltage DC multipliers. IEEE Trans. Power Electron..

[21] Bellar M.D., Watanabe E.H., Mesquita A.C. (1992). Analysis of the dynamic and steady-state performance of Cockcroft-Walton cascade rectifiers. IEEE Trans. Power Electron..

[22] Kobougias I.C., Tatakis E.C. (2010). Optimal design of a half-wave Cockcroft–Walton voltage multiplier with minimum total capacitance. IEEE Trans. Power Electron..

[23] Maennel C.G.H. (2013). Improvement in the modelling of a half-wave Cockcroft-Walton voltage multiplier. Rev. Sci. Instrum..

[24] Weiner M.M. (1969). Analysis of Cockcroft-Walton voltage multipliers with an arbitrary number of stages. Rev. Sci. Instrum..

[25] Sutmann G. (1998). Structure formation and dynamics of water in strong external electric fields. J. Electroanal. Chem..

[26] Zangi R., Mark A.E. (2004). Electrofreezing of confined water. J. Chem. Phys..

[27] Zangi R., Mark A.E. (2003). Monolayer ice. Phys. Rev. Lett..

[28] Zangi R. (2003). Bilayer ice and alternate liquid phases of confined water. J. Chem. Phys..

[29] Koga K., Tanaka H. (2005). Phase diagram of water between hydrophobic surfaces. J. Chem. Phys..

[30] Bai J., Angell C.A., Zeng X.C. (2010). Guest-free monolayer clathrate and its coexistence with two-dimensional high-density ice. Proc. Natl. Acad. Sci. U. S. A..

[31] Jinesh K.B., Frenken J.W.M. (2008). Experimental evidence for ice formation at room temperature. Phys. Rev. Lett..

[32] Khusnutdinoff R.M., Mokshin A.V. (2019). Electrocrystallization of supercooled water confined by graphene walls. J. Cryst. Growth.

